# Is There a Need for a More Precise Description of Biomolecule Interactions to Understand Cell Function?

**DOI:** 10.3390/cimb44020035

**Published:** 2022-01-21

**Authors:** Pierre Bongrand

**Affiliations:** Lab Adhesion and Inflammation (LAI), Inserm UMR 1067, Cnrs UMR 7333, Aix-Marseille Université UM 61, Marseille 13009, France; pierre.bongrand@inserm.fr

**Keywords:** systems biology, artificial intelligence, lymphocytes, catch bonds, bond strengthening, molecular dynamics

## Abstract

An important goal of biological research is to explain and hopefully predict cell behavior from the molecular properties of cellular components. Accordingly, much work was done to build extensive “omic” datasets and develop theoretical methods, including computer simulation and network analysis to process as quantitatively as possible the parameters contained in these resources. Furthermore, substantial effort was made to standardize data presentation and make experimental results accessible to data scientists. However, the power and complexity of current experimental and theoretical tools make it more and more difficult to assess the capacity of gathered parameters to support optimal progress in our understanding of cell function. The purpose of this review is to focus on biomolecule interactions, the interactome, as a specific and important example, and examine the limitations of the explanatory and predictive power of parameters that are considered as suitable descriptors of molecular interactions. Recent experimental studies on important cell functions, such as adhesion and processing of environmental cues for decision-making, support the suggestion that it should be rewarding to complement standard binding properties such as affinity and kinetic constants, or even force dependence, with less frequently used parameters such as conformational flexibility or size of binding molecules.

## 1. Introduction: Why Is There a Need for Elaborate Parameters to Describe the Properties of Cellular Components?

A long-term goal of cell biologists consists of explaining cell function with basic laws of physics and chemistry [1,2]. This requires to address the following questions:

(i) Define and identify informative cell **properties** (i.e., features or attributes). The choice of considering a particular property is somewhat arbitrary and depends on the function that is being explored. As an example, the set of gene transcription rates may be thought to account for a cell differentiation state [3]. More transient properties are cell polarization, i.e., asymmetrical organization, or motility state: whether a cell is immobile on a surface or migrating throughout a living organism. The activity of a particular signaling pathway is another example. A cell **state** may be defined as the time-dependent set of values of a suitable group of properties.
(ii) Define and identify the pieces of information, or **signals**, provided to cells by the extracellular medium. A signal may consist of the application of a force on a region of the cell membrane or binding of a ligand to a cell surface receptor. As will be discussed below, multiple binding interactions are continually formed and broken, and a choice may be necessary to define **significant signals**, which depends on the function that is being explored. Additionally, the effect of a signal may be strongly dependent on its localization and temporal evolution. These features must, therefore, be included in the **parameters** used to describe signals.
(iii) Discover **rules** allowing to predict the temporal evolution of cell states as a function of received signals. It must be emphasized that the way rules are expressed may involve somewhat hidden assumptions. Thus, it may be implicitly assumed that the description of a cell state does not need to include its history, i.e., that cell evolution may be described as a Markov process, an assumption that is not always warranted [4,5].

Thus, the first step of analyzing a cell function consists of selecting and defining **parameters** that can be measured and processed and that will provide a suitable description of cell properties. As an example, the property of a molecule A to bind to another molecule B may be described by using the affinity constant, which is a quantitative parameter. A property may also be described in a binary (or Boolean) way: when a couple of molecules A and B is considered, it is described as “physically interacting” or not. The purpose of this review is two-fold. Firstly, we shall briefly discuss the dependence of different methods used to study cell functions on the initial choice of the parameters. Secondly, we shall focus on the description of biomolecular interactions, since they are considered as prominent contributors to cell function, and we shall ask whether the parameters currently used to describe molecular interactions are sufficient to allow the tools currently used by cell biologists to elucidate cell behavior. 

## 2. The Initial Choice of Parameters Used to Describe Cell Properties Strongly Influences the Performance of Quantitative Methods Currently Used to Study Cell Function

Before focusing on the description of biomolecule interactions, it seemed useful to rapidly discuss the power and limitations of strategies currently used to model cell function within the domain of systems biology. Indeed, the information that can be obtained on cell function is dependent on the choice of parameters that can be defined and measured, and the choice of methods that will be used to process data. We shall describe several representative examples of strategies that may be considered to study cells or cellular components.

### 2.1. An Exhaustive Ab Initio Description of Cell Function Seems out of Reach in the near Future, and It Is not as “Parameter-Independent” as Might Be Thought: Lessons from Molecular Dynamics

Computer simulation is a powerful way of studying complex systems. Molecular dynamics (MD) is a prominent example and it brought new information on protein structure and biomolecule interactions [6,7]. A brief discussion of the basic principles, recent advances, and limitations seems warranted to discuss the relevance of this approach to cell function. See, e.g., [8] for a technical description of Gromacs, a freely available MD software providing a good example of currently performed MD simulations [9,10].

A typical MD computation consists of simulating the evolution of a protein molecule in a box filled with water and ions. The current orders of magnitude are 100 nm for the linear size of the box, 10,000 for the number of atoms in the protein, and 100,000 for the number of water molecules or number of water atoms. A full description of the protein may, thus, involve an order of 60,000 parameters, corresponding to the positions and velocities of all atoms. The simulation consists of starting from a reasonable conformation, usually on the basis of a structure obtained by an experimental method such as X-ray crystallography, and calculating the forces experienced by all atoms with empirical force fields in order to determine the displacement and velocity changes of individual atoms during a time step on the order of 1 fs (10^−15^ s). This procedure is then repeated for at least several millions of steps, with periodic recording of the parameters, yielding “trajectory files” that can be processed to determine requested pieces of information. MD has been used for more than a decade to obtain insight on ligand-receptor interactions [6]. This approach is now widely used to address a variety of problems, such as an estimate of binding affinities [11], check of the quality of experimentally determined structures [12], or assessment of the influence of the conformational variability of proteins on docking behavior [13]. This strategy was, thus, able to bring new insight into protein structure and function without any a priori model of investigated phenomena.

However, applying this powerful approach to whole cells is clearly out of reach: the size of a typical cell is on the order of 10 µm, and the number of atoms is, thus, 15^15^-fold higher than accessible with current MD simulations. The number of steps required to reach physiologically significant time periods would need a more than 1000-fold increase. As recently emphasized [2], the computational power required to deal with this complexity is not expected to be available before many decades. Therefore, a drastic dimensional reduction would be required to simulate cellular systems. Noticeably, such simplification was already required to explore physiologically relevant properties of single proteins or ligand-receptor couples with MD. So-called coarse-grained simulations might consist of replacing individual atoms with groups such as water molecules [14], accelerated sampling [15,16], splitting long simulations by a number of shorter ones with different starting configurations (so-called umbrella sampling), or increasing transition rates with well-chosen virtual forces (steered MD) allowed to explore more extensive regions of conformational landscapes.

As was recently emphasized [17], a conventional simulation can now generate terabytes of data, and data processing requires elaborate methods that are not always easy to fathom, and the outcome of which may depend on the choice of parameters used to describe studied systems. As an example, it was shown with a toy model that unsupervised “machine learning” tools failed to identify important features of a system described with cartesian coordinates rather than interatomic distances [17].

While a more detailed discussion of MD would not fit into the scope of this review, this example clearly shows that any attempt at a quantitative understanding of cell function should require for many years a huge simplification and a choice of a restricted set of parameters that may strongly influence the outcome of modeling attempts.

### 2.2. The “Omic” Approach: Representative Examples of Strategies Used to Analyze Huge Datasets

During the last two decades, following the completion of the human genome project, much effort was made to build extensive datasets of key cell properties, such as the status of gene transcription (transcriptome [3]), protein content (proteome [18,19]), intracellular biochemical reactions (metabolome [20]), or list of molecular interactions (interactome [21,22]) and computational tools were developed to process available data. Not surprisingly, the power and limitations of data processing tools are highly dependent on the precise parameters that are fed into databases. We shall give a few general examples before focusing on parameters used to account for biomolecule interactions in Section 3.

#### 2.2.1. Graph Theory, Networks, and Logic-Based Models

**Figure 1:**
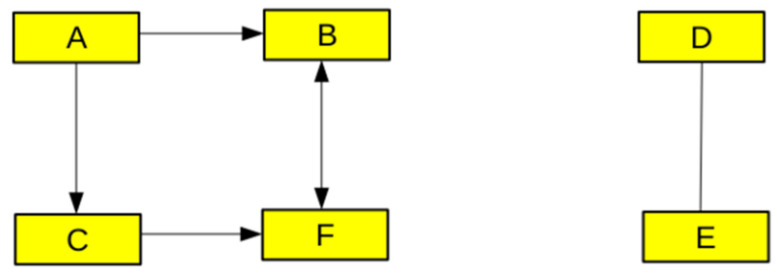
Example of a network representation of six molecules (A, B, C, D, E, F) that may be, e.g., enzymes or cytoskeletal components. The edges may represent binding interactions, or enzyme activation and inhibition. Network analysis may reveal remarkable patterns, also called motifs, that can be ascribed a particular function (such as retroinhibition) [27]. A relatively independent group (such as ABCF) may be viewed as a fairly autonomous module and represented by a single node for network simplification. Additionally, the frequency of occurrence of a particular motif such as ABCF in a huge network can be calculated, and this may be compared to the frequency expected with a random distribution of edges [25].

Networks, which are also called graphs in the mathematical literature, may be defined as collections of points (also called nodes or vertices) joined in pairs by lines (also called edges or links) [23]. This basic principle is displayed in Figure 1. They are widely used in the biological literature to summarize large datasets. As an early example, an analysis of inflammation was performed by using microarrays to measure the transcription rate of 3714 genes in blood leukocytes of human volunteers before and 2, 4, 6, 9, and 24 h after intravenous injection of an inflammatory stimulus [24]. A statistical analysis was performed to determine the genes that displayed increased, decreased, or constant transcription rate. A computer-assisted analysis of over 200,000 scientific papers was performed to identify connections of different types (e.g., physical or transcriptional) between gene pairs. The results were displayed as a network involving 1556 genes and their interactions. Each gene was represented as a node with three discrete states. In a similar spirit, integrin-mediated cell adhesion was analyzed by searching published data. The results were represented as a network made of 156 nodes representing so-called adhesome components and 690 interactions that might consist of physical interaction, activation, or inhibition [25]. Network analysis was shown to yield valuable hints on the function of displayed molecules [26,27]. Obviously, this analysis is strongly dependent on the choice of parameters represented by the edges.

This representation is a good starting point for the building of a Boolean network as a basis of dynamic modeling of cell function [28,29,30]. This strategy was used to analyze the differentiation of CD4+ T lymphocytes that play a key role in orchestrating immune responses [29]: following stimulation with infectious agents, CD4+ may differentiate into different subtypes that will preferentially activate a particular branch of the immune response. Thus, Th1 cells will enhance cellular effectors by activating cytotoxic T cells or mononuclear phagocytes. Th2 cells will activate humoral responses. Treg cells will prevent excessive activation and autoimmunity. The balance between possible differentiation pathways may determine the outcome of an infection. Thus, intracellular pathogens are known to escape antibody responses. It is, therefore, of prominent medical importance to predict and possibly manipulate the outcome of a particular stimulation. The authors scanned the scientific literature to build a regulatory network including 18 nodes (cytokines or transcription factors) with two possible states (active or inactive) and Boolean algebraic equations based on known biological data to obtain dynamical predictions. As an example, the state of IL2-receptor at time t + 1 was calculated as {(IL-2 at time t) AND (NOT SOCS-1 at time t)}, (IL-2 is interleukin 2, SOCS is suppressor of cyokine synthesis). A notable finding is that this approach yielded so-called **attractors** (as defined within the framework of chaos theory), i.e., states that remain stationary and that were concluded to correspond to already-known differentiation states. More recently, a similar strategy was used to analyze the differentiation pathways followed by macrophages [31]. The Boolean model comprised 29 nodes (such as cytokines or kinases) and 60 interactions. This was aimed at better controlling the balance between pro- and anti-inflammatory fates.

These simple examples deserve some comments: (i) as was early emphasized, the use of logic-based models made it possible to perform a dynamic treatment of complex systems in absence of detailed quantitative experimental data [28,30]. (ii) This approach is appealing since it is fairly easy to grasp intuitively. (iii) The validity is difficult to assess rigorously, and it is not obvious to determine which features of a network may be considered as unique. (iv) The building of the network is based on the use of arbitrary thresholds to achieve a binary description of molecular states and interactions [29]

Therefore, it is not surprising that more quantitative models were considered to be necessary to account for complex biological systems. Thus, fuzzy logic was proposed as a more quantitative approach, with a possibility of more than two states of a node, and probabilistic transitions [32]. Boolean models were transformed into quantitative models in order to make use of quantitative experimental data, and this approach was applied to a study of T lymphocyte signaling [33]. However, a detailed discussion of these attempts would not fall into the scope of the present review.

#### 2.2.2. Viewing Cells as Mobile Points Moving on a Multidimensional Landscape

The starting point was a highly quoted metaphor by C. Waddington, who compared differentiating cells to marbles rolling down a surface with valleys (corresponding to developmental paths) and local minima [34,35]. This general representation recently met with considerable success. In the widely used Pubmed database, the number of papers retrieved with the “landscape” keyword increased from 269 to 10,468 (0.7% of published papers) between the years 2000 and 2020. As an example, the reprogramming of mouse embryonic fibroblasts into pluripotent stem cells was studied with single-cell RNA sequencing [3]. A total of 315,000 cell samples were sampled at short time intervals during an 18-day period and the cell trajectories in the developmental landscape were derived with a mathematical procedure (so-called optimal transport theory) by relying on the assumption that cells displayed straightforward displacement in a short time, and the displacement probability did not depend on displacement history (following a Markov model). A theoretical model of epithelial-to-mesenchyme transition was built on the basis of 16 representative genes previously identified in experimental studies [36]. The landscape metaphor was also used to account for different biological processes, such as the signaling pathways of T lymphocyte activation, as revealed by analyzing cell proteome and phosphoproteome [37], the immunological status of infected patients as a point in a 19-dimensional space representing 19 blood cell types [38], or cell shape control by a couple of interacting Rho GTpases [39]. More mathematically oriented reports aimed at showing that the dynamics of complex systems were amenable to such a geometrical representation [40,41].

This brief description deserves two remarks: (i) using models that were thoroughly studied in other fields of science has long proved a very fruitful approach in the domain of mathematics and physics. (ii) It may be difficult to assess the significance of parameters that are fed into these models. Thus, epithelial-to-mesenchyme transition was modeled [36] with a network involving 16 nodes representing genes or micro-RNAS, the relevance of which was demonstrated experimentally. Activation, inhibition, and interactions were modeled with functions involving a number of parameters, such as degradation rates (16 parameters) or mutual activation (16 × 15 = 240 parameters are needed to account for the effect of each component on the 15 others). In order to make the model manageable, a number of parameters were slumped together, resulting in a total of five parameters. Thus, while the model could yield metastable states and some path transitions consistent with experimental data, it is very difficult to determine the actual biological significance of nodes and activation parameters.

### 2.3. Data Processing with Multivariate Statistics and Machine Learning

Multivariate statistics [42] have long been used to analyze large biological datasets [43,44] and perform tasks such as identification of important parameters (with techniques such as principal component analysis), significant groups in large sets of points (with so-called clustering techniques), or correlations between different parameters and prediction of outputs from input parameters. Discrete data, such as DNA sequences, are particularly amenable to these powerful methods, and user-friendly tools were developed to make them accessible to a wide community of users [45]. Furthermore, during the last decade, artificial intelligence was more and more successfully used to perform complex tasks due to the progress brought by deep learning to conventional machine-learning technology [46,47,48]. This met with impressive success in a wide variety of fields, including image analysis [49], medicine [50], and protein studies [51]. It is, therefore, not surprising that machine learning is more and more often used to analyze cell processes such as immune cell differentiation [52] or relate DNA sequence to cell morphological patterns [53].

However, despite impressive successes met by artificial intelligence in finding patterns in complex datasets and predicting outputs, some unanswered questions hamper their contribution to our understanding of cell function. First, the complexity of trained networks caused them to be considered as black boxes, and attempts are currently done to decrease their opacity [54,55] or increase reliability [56,57]. Secondly, a well-known caveat of correlation studies is that a strong correlation between two parameters is not a proof of a causal relationship between these, and unveiling causal relationships is by no means a simple task [58]. This limitation is important because it may hamper the understanding of a cellular process if the parameters used to describe this process are not directly involved in its mechanism. Thus, the very efficiency of machine learning to find patterns in complex datasets makes it difficult to conclude that the parameters used to measure the studied phenomena were **directly** and **causally** related to cell function.

*In conclusion*: as shown by the few examples we described, much effort is currently being made in gathering huge datasets to describe various cell functions, and highly sophisticated methods are currently developed to try and interpret these data and identify hopefully predictive rules. The significance of conclusions is dependent on the choice of parameters used to describe experimental data. The growing complexity of currently published papers makes it more and more difficult to assess the validity of models, and intrinsic relevance of chosen parameters. In the following section, we shall try to clarify this, admittedly too general and abstract, statement by considering a more specific question: which parameters should be used to account for protein interactions in order to derive optimal benefit from the enormous amount of available information.

## 3. Is There a Need for a More Precise Description of Biomolecule Interactions?

### 3.1. Current State of Interactome Databases

It has long been recognized that biophysical interactions between cell components play a key role in the cell structure and function [6,21,59,60]. Cell cohesion obviously needs stable attachments between structural molecules. Cell communication with the outer world relies on the adhesion to other cells or surfaces, and recognition of soluble or surface-bound mediators with hundreds of membrane receptors [61]. Generation and propagation of intracellular signaling events relies on the formation and dissociation of multimolecular complexes [62,63]. Molecular transport throughout cells is strongly influenced by interdependent crowding and binding effects. Thus, much effort is currently being made to gather extensive experimental information on biomolecule interactions and particularly protein interactions, to build extensive maps of the so-called **interactome**. While a detailed description of the current state of this highly moving field would obviously fall outside the scope of this review, it is important to emphasize some points:

(i) *The term “interaction” may refer to different phenomena*. Physical binding and unbinding of a molecular pair in solution can be quantified with high accuracy with standard techniques, as will be detailed below. High throughput maps of binary interactions involving about 17,500 human proteins (about 90% of the protein-coding genome) were built with the standard *yeast two-hybrid method*, yielding about 53,000 interactions [60]. The validity of results was fed into public databases such as IntAct (https://www.ebi.ac.uk/intact/ accessed on 7 December 2021) after careful validation [60]. However, as was well acknowledged by the authors, the occurrence of physiologically relevant molecular interactions requires that molecular partners might encounter each other within cells. Additionally, molecular interactions may display significant differences in vitro and within cells [64]. *Affinity purification-mass spectrometry* (AP-MS) is currently used to obviate this difficulty [65,66]. This relies on the use of cells expressing tagged “baits” that may be purified after cell lysis before identification of binding partners with quantitative mass spectrometry. As a recent example, a network of 118,162 interactions among 14,586 proteins was obtained after affinity-purification of 10,128 proteins expressed by human epithelial kidney cells, yielding the Bioplex 3.0 network [21]. Interestingly, comparison with data obtained on another cell line (from human colorectal carcinoma) revealed, as expected, significant differences between interatomic networks found with two different cell types. Additionally, when AP-MS was studied to monitor, for a period of time of 600 s, primary T lymphocytes subjected to antigen-receptor mediated stimulation, the expected evolution of signalosome interactions was clearly evidenced [65]. *In conclusion*, while a reliable and nearly exhaustive network of the physical interaction of human proteins under standard conditions may be available, an exhaustive description of interactions involving relevant epigenetic states of the proteome and cellular environments is currently out of reach [21,60], despite the impressive amount of information gathered by combining data mining and experiments, as exemplified by the STRING database [22]. As clearly stated [60]: “It remains infeasible to assemble a reference interactome map by systematically identifying endogenous protein–protein interactions (PPIs) in thousands of physiological and pathological cellular contexts”.
(ii) As already mentioned in the aforementioned examples, the quantitative properties of biomolecule interactions may display huge variations. As an example, the dissociation constant of biomolecular bonds may vary between picomolar values (as exemplified by hormone-receptor interaction) and millimolar values [67], which may be considered to represent ultra-weak interactions, of which the biological importance is, however, recognized [68]. The lifetime of a ligand-receptor bond may vary between less than a second (as was sometimes reported on cadherins [69] or antigen-antibody pairs [70]) and hours.

*In conclusion*, recent work has provided scientists with an extensive description of biomolecular interactions that were represented as essentially qualitative networks. This situation may be illustrated by a search made on several well-known databases to find information on the interactome of P-selectin, an endothelial adhesion molecule that was shown to mediate the rolling of blood leukocytes on inflamed endothelium by binding to its main ligand PSGL-1 with a high binding rate and resistance to forces [71,72]. As shown in Table 1, the P-selectin ligand was easily retrieved, but the specific quantitative features of receptor–ligand interactions were not immediately displayed. The analysis of these networks with suitable algorithms yielded new information on, e.g., signaling pathways [37,65]. An open question is to know whether this basis may be sufficient to allow a quantitative understanding and prediction of cell behavior provided suitable processing methods are developed, or whether there is a need to incorporate a number of other quantitative parameters in datasets. While it is certainly not possible to give a definitive answer to these questions, it is certainly useful to review parameters recently used to account for biomolecule interactions and discuss their relevance to cellular processes.

### 3.2. Parameters Currently Available to Describe Biomolecule Interactions

In this section, we shall briefly describe methods currently available for quantitative studies of ligand–receptor interaction and parameters yielded by these methods. In the next section, we shall discuss the need of using these parameters to unravel cell function. 

Here, our purpose is not to present an exhaustive description but only to give a feeling for the kind of information that is currently available. 

#### 3.2.1. Interaction between Soluble Molecules and Surface-Bound Receptors

**Figure 2:**
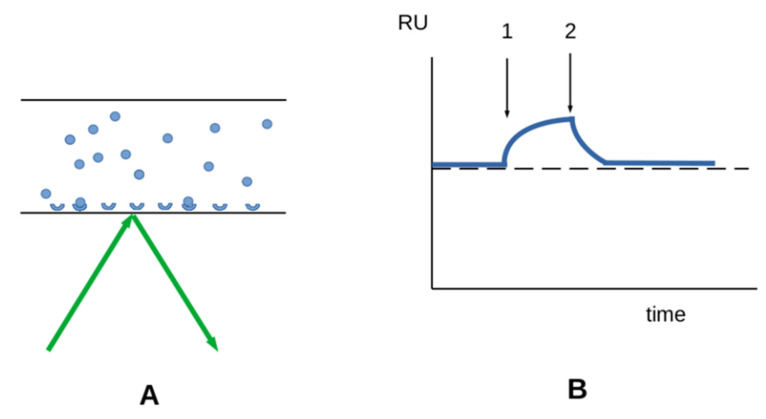
**Studying ligand-receptor interaction with SPR.** (**A**) A ligand solution (blue disks) is driven through a narrow channel, of which the floor is coated with receptor molecules. A laser beam (green arrows) is used to probe the optical properties at the interface, allowing real-time determination of the amount of bound material. (**B**) A typical curve. After an equilibration period, the ligand solution is driven into the channel, resulting in progressive increase of bound material (first arrow). The ligand solution is then replaced with an empty buffer and the ligand release is monitored. The on- and off-rate can, thus, be determined. A significant cause of error is the presence of molecular aggregates in the ligand solution.

*Surface plasmon resonance (SPR)* is a widely used method [73,74]. A protein solution is driven along a receptor-coated surface in a narrow channel and the amount of bound material is determined in real time by probing the refractive index near the surface with an evanescent wave. When equilibrium has been reached, the protein solution is replaced with an empty buffer and the kinetics of protein release is determined. This method allows label-free determination of the kinetic constants of bond formation and dissociation (Figure 2), and the equilibrium constant can be calculated as the ratio between these kinetic constants (Section 3.3). In contrast, standard radioimmunoassay (RIA) or enzyme-linked immunosorbent assays (ELISA) only yield equilibrium constants. It is usually considered that the mechanisms of interaction between a soluble and a surface-bound molecule are comparable to the interaction between two soluble molecules; however, they are easier to study. Another point is that the measured parameters represent average values of a large number of molecules. The receptor and solute must be homogeneous.

#### 3.2.2. Interaction between Surface-Bound Ligands and Receptors

The aforementioned methods are subject to two kinds of limitations (see [61] for a recent discussion). First, important cell functions such as adhesion or migration on surfaces are mediated by membrane receptors and surface-bound molecules. Second, as will be described in Section 3.3.4, kinetic rates of bond formation and dissociation are not sufficient to account for all important cell phenomena. These points are important since the properties of interactions between surface-bound molecules (two-dimensional or so-called 2D interaction) cannot be derived from data obtained with soluble molecules (so-called 3D interactions) [75]). We shall briefly describe three methods that were widely used to study 2D interactions during the last two decades [70,76].

**Figure 3:**
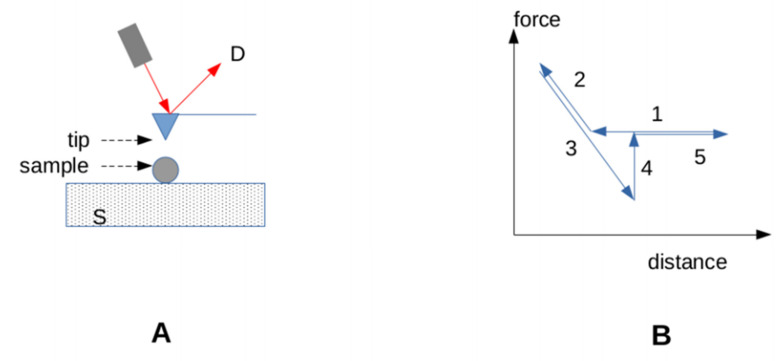
**Atomic Force microscopy**. (**A**) The ligand-coated tip (blue triangle) is bound to a flat spring (cantilever), bearing a mirror that reflects a laser beam (red arrows) towards a detector (D), allowing rapid and sensitive determination of vertical displacements. The receptor-bearing sample is deposited on a receptor-coated block (dotted surface S) driven by a piezoelectric device that is moved up and down. (**B**) Real-time determination of the force applied on the tip. Contact is achieved during an upward move (2), and the surface is, thus, lowered, resulting in a progressively increasing force (3) until the bond is broken (4).

*Atomic force microscopy (AFM)* was very early used to study strong interactions, such as streptavidin-biotin or antigen-antibody bonds. As depicted in Figure 3, the ligand-coated nanometer-width tip of an AFM is pushed against a receptor-coated surface with a typical force of order of 50–100 pN and a typical duration of order of 100 ms. The tip is, thus, subjected to a disruptive force increasing with a constant rate called the **loading rate**, expressed in pN/s. This setup was shown to evidence single-bond attachments that were usually broken when the applied force ranged between a few tens of piconewtons [77,78] and nearly 200 pN [79]. The measured value is called the unbinding force. Several limitations must be mentioned: first, the unbinding force is not an **intrinsic** molecular parameter, since it is dependent on the loading rate. Second, the kinetic constant of bond formation is difficult to estimate, since the contact area cannot be measured accurately, and the dependence of the binding probability on contact duration is difficult to estimate. Third, the monitoring of very weak bonds, such as the interaction between T cell receptors and their ligand, may be difficult to achieve [80].

**Figure 4:**
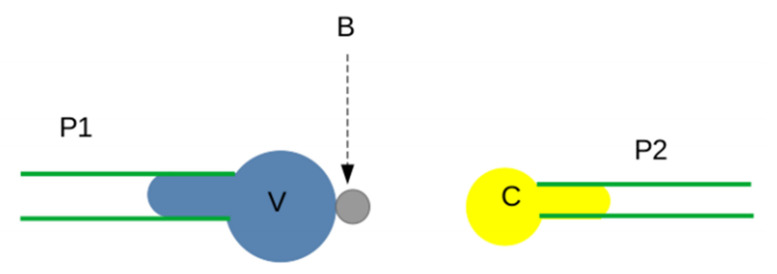
**Biomembrane force probe**. A ligand-derivatized microsphere B (gray disk) is glued to a soft vesicle, such as an erythrocyte (V), and sucked with controlled pressure into a micropipette (P1). A receptor-bearing cell C is held with a piezoelectric-driven micropipette (P2) and driven against the microsphere. The force is derived with high sensitivity from the microsphere displacement, since the vesicle acts as a soft and tunable spring.

*The biomembrane force probe (BFP)* allowed to increase the sensitivity and resolution of force studies performed with the AFM. The basic principle consisted of replacing the AFM cantilever with an inflatable vesicle that might be viewed as a tunable spring, as depicted in Figure 4. This allowed to vary the loading rate over a very wide range of nearly 10 orders of magnitude. The analysis of the dependence of unbinding force on loading rate, later dubbed “dynamic force spectroscopy”, yielded some information of the shape of the energy/distance curve of the ligand receptor complex [81]. This point will be discussed below. 

**Figure 5:**
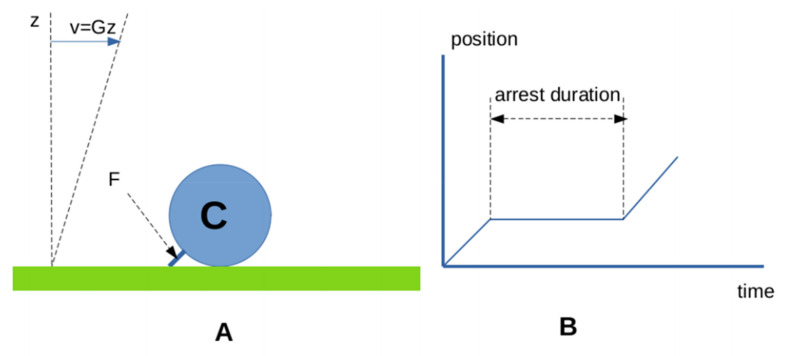
**The laminar flow chamber**. (**A**) a receptor-derivatized microsphere (blue disk) is driven along a surface coated with very low ligand densities with a low shear laminar flow. The hydrodynamic force on a bond maintaining the microsphere at rest may be as low as a few piconewtons [82]. An automated tracking system yields a plot of cell movement (**B**). Periods of constant velocity displacements are interspersed with arrests of varying duration. Arrest frequency and duration are tightly related to the molecular rate of bond formation and dissociation.

*The laminar flow chamber* (LFC, Figure 5) has been used for nearly three decades to study the formation and rupture of single molecular attachments between surface-bound molecules. This technique was extensively described in a previous review [82] and important results obtained with the LFC were recently reviewed [61]. Briefly, receptor-bearing cells or microspheres are driven along surfaces sparsely coated with ligand molecules with a low shear hydrodynamic flow, which imparts to them a typical velocity on the order of tens of µm/s. Particle movement is tracked automatically with a system typically yielding a space and time resolution of tens of nm for position (if particles are microspheres) and 20 ms (using standard video cameras), respectively. A single molecular bond is sufficient to stop the particle since the applied hydrodynamic force may be as low as a few piconewtons. The duration of contact between ligand and receptor molecules may be estimated as a function of molecular length. Varying the flow velocity allows to derive quantitative relationships between the contact duration, force on the bond, binding frequency, and distribution of arrest duration.

The laminar flow chamber provided an efficient means of obtaining large statistics on the formation and dissociation of weak bonds with a subsecond lifetime. A common problem with AFM, BFP, and LFC is to ensure that monitored binding events are indicative of single molecular bonds [83]. This is easily achieved with LFC by checking that sequential dilutions of the density of binding sites on the chamber floor result in decrease of bond formation without bond lifetime alteration.

#### 3.2.3. Additional Parameters May Be Obtained with Computer Simulation

The continuous improvement of molecular dynamics in the last few decades [84] has made it possible to obtain extensive information on the formation and rupture of bonds between proteins subjected to controlled forces that were introduced at will as constraints. While it is not yet feasible to monitor a molecular complex for several seconds, clever tricks, such as *steered molecular dynamics* or *umbrella sampling*, made it possible to obtain accurate information on the conformational changes and intermediate states displayed by receptors and ligand molecules during the binding and unbinding process. This provided a highly valuable help to achieve a quantitative understanding of molecular interactions. However, the very abundance of experimental and simulated data [17] now raises two important questions: (i) which set of parameters can give a sufficiently exhaustive and still manageable account of the binding process and (ii) which information is actually needed to achieve a reasonably accurate understanding of cell function. These key points, which are dependent on available tools for data gathering and data processing, will be considered in the following section. Interestingly, the insufficient incorporation of structural information in systems biology was recently emphasized [85]

### 3.3. Physical and Biological Significance of Parameters Allowing to Account for Biomolecule Interactions

#### 3.3.1. The Equilibrium Constant

The law of mass action has long been considered as a fundamental basis for understanding atomic and molecular association, and it was the subject of whole books [86]. Indeed, three decades ago, the concept of affinity was considered to dominate most thinking about complex biological reactions [87]. It is well known that when two molecular species A and B forming a complex AB are mixed and left to reach equilibrium, the final concentrations of A, B, and AB are related by the following simple equation:

[AB]_eq_/[A]_eq_·[B]_eq_ = K_a_ = 1/K_d_ (1)

where K_a_ is the **affinity constant** and K_d_ the **dissociation constant**, which are usually expressed in M^−1^ and M, respectively. K_a_ is close to the (dimensionless) quantity exp(-ΔF°/RT), where ΔF° is the standard free energy, R is the perfect gas constant, and T is the absolute temperature (see [70] for details). Importantly, ΔF° contains a term accounting for the structural reorganization of A and B during complex formation and a so-called connection term accounting for the loss of some degrees of freedom of A and B (such as translational and rotational motion) after bond formation.

The dissociation constant K_d_ of biomolecular bonds may vary over more than ten orders of magnitude (from millimolar values reported on ultraweak interactions [67] to femtomolar affinity of the streptavidin-biotin couple [70]). It might seem reasonable to hypothesize that there is a need to know the affinity of reported interactions to understand their function. However, the utility of quantitative affinity data is hampered by two important limitations.

1. It is difficult to use Equation (1) to assess the biological significance of a reported interaction. Indeed, it would reasonable to conclude that an interaction AB is significant if either K_a_[A] or K_a_[B] is not too close to zero. But this would require to know the value of [A] close to a molecule [B] or [B] close to [A], which is not always the case. As a striking example, it was recently shown that spatio-temporal cAMP signaling is under precise control of nanoscale domains [88]. Additionally, local molecular crowding may alter effective concentrations [89]. More generally, the in vivo affinity of a reaction may differ significantly from the affinity measured in a standard buffer. Thus, the qualitative demonstration of a physical interaction between two molecules within a cell as evidenced with AP-MS [21] may be more informative than quantitative in vitro affinity measurement.
2. Many biomolecule interactions involve surface-bound molecules. Indeed, as was recently emphasized [90], nearly 30% of human genes encode membrane proteins. Unfortunately, as already explained [75], the affinity between surface-bound molecules is difficult to derive from 3D affinity, and even to define: the outcome of an encounter between two bound molecules is dependent on a number of parameters independent of binding sites such as molecular length and flexibility [91], lateral mobility, and distance between surfaces [92].

*In conclusion*, the **affinity constant** may not be the most useful parameter to assess the influence of a given interaction on cell function.

#### 3.3.2. Kinetic Rates Provide an Informative Means of Accounting for Biomolecule Interactions

The simplest way of describing the time-dependence of association between molecules A and B is to use the well-known two-parameter equation [70]:

d[AB]/dt = k_on_ [A] [B] − k_off_ [AB] (2)

where the rate of bond formation (on-rate) k_on_ and the rate of complex dissociation (off-rate) k_off_ are related to the equilibrium constants as:

K_a_ = k_on_/k_off_; K_d_ = k_off_/k_on_ (3)

As was recently reviewed [61], this kinetic description provided a substantial help to the analysis of complex biological phenomena. An early example is the attachment of blood leukocytes to the vessel walls as an early step of inflammation. This was found to proceed as a two-step phenomenon. Leukocytes first move along the inflamed endothelium with a jerky motion called rolling, and then stop. Rolling is essentially mediated by selectin molecules, whereas integrins make cells stop. This difference was ascribed to a rapid binding kinetics of selectins as compared to integrins [71]. Another example is the triggering of immune responses by T lymphocytes detecting a foreign antigen such as a viral protein exposed by an infected cell [93]: the task of a lymphocyte consists of recognizing an antigen out of a few tens of copies of complexes (pMHC) made of a major histocompatibility complex molecule (MHC) and a viral oligopeptide (p) that is buried among nearly 100,000 pMHCs differing by only a few amino-acids. Detection must occur during an intercellular contact lasting only few minutes, and the detection specificity was claimed to approach the physical limits of the specificity of molecular interactions [93]. An early finding was that the lifetime of pMHC interaction with T lymphocyte receptors (TCRs) was a key parameter for recognition. These two examples support the intuitive assumption that kinetic parameters may help us determine the significance of a molecular interaction. Stable bonds may be expected to play a key role in cell structure. Transient interactions are expected to drive the diffusion of intracellular molecules, signal formation and propagation, cell displacement, and detection of specific molecular patterns on neighboring surfaces.

However, the biological information provided by the on-rate and the off-rate is hampered by two difficulties:

1. First, biomolecular attachments often appear as multistep reactions, the description of which may require numerous parameters to account for several energy barriers in reaction paths. The number of required parameters was somewhat reduced when it was found that the kinetics of bond formation between different antigen and antibody might be modeled as a progression along a rugged energy landscape, accounted for by a single effective diffusion constant, and matched the intuitively appealing interpretation that bond formation required a minimal contact time that was estimated as a few milliseconds [94,95,96]. Thus, in contrast with the predictions of Equation (2), a fairly long contact may not always be replaced with many transient ones to allow bond formation.
2. Second, the on-rate is difficult to define under 2D conditions, since it depends on many properties independent of the molecular binding interface [75,92]. Additionally, the bond lifetime is certainly as dependent on the disruptive forces exerted by surfaces, as it depends on the intrinsic bond stability [97].

*In conclusion*, while there is an absolute requirement for some information on the kinetic properties of a biomolecule interaction to understand its biological function, there remains to determine if the use of the on-rate and the off-rate is sufficiently exhaustive to contain this information. In particular, it might be of interest to explore the predictive value of the minimal time of bond formation we have just mentioned [96].

#### 3.3.3. Accounting for the Effect of Forces on Bonds

As indicated in the previous section, while the off-rate of a bond involving soluble molecules under standard conditions may be considered as a well-defined parameter, the rupture of a bond formed between surface-attached molecules is strongly dependent on the time dependence [81] and direction [98] of applied forces. This is important since cells continually apply forces on many molecules, including membrane receptors involved in adhesion, such as integrins, or communication, such as notch [99,100], T cell receptors [101], and cytoskeleton-associated molecules [102]. Understanding cell function, therefore, requires a suitable description of the effect of forces on molecular bonds. A number of parameters were used for this purpose.

The **bond strength**, i.e., the force needed to “rapidly” break a bond seemed a convenient parameter when this was studied with experimental methods described in Section 3.2.1. Reported orders of magnitude varied between a few tens of piconewtons, as exemplified by T cell receptors [80], and hundreds of piconewtons, as exemplified by selectins [79]. An exceptionally high adhesive strength of over 2000 pN was reported for a bacterial adhesin [103]. 

The problem is that while bond strength is an appealing parameter, it is not well defined. As was clearly noted by George Bell in a widely quoted paper [104], bond rupture is a random phenomenon, the frequency of which depends on applied forces. Bell suggested the following formula, which is often referred to as Bell’s equation [105,106]:

k_off_(F) = k_off_(0) exp (*a*F/k_B_T) (4)

where k_off_(F) is the off-rate of a bond subjected to a pulling force F, k_B_ is Boltzmann’s constant, T is the absolute temperature, and *a* is a parameter with the dimension of a length. This empirical formula can be made intuitive with a very naïve model: bond rupture may be viewed as the exit of a particle from an energy well. The exit probability may be approximated as the frequency of attempts to exit multiplied by the probability of success, which is expected to be close to the probability that the particle energy is higher than the energy barrier W. The probability of success should thus be proportional to exp(-W/k_B_T), according to Boltzmann’s law. A force F is expected decrease W by the product *a*·F, where *a* is the distance between the energy barrier and the resting position. This simple law was found to give a satisfactory account of effect of force on selectin-mediated bonds [105], and the results obtained on other ligand receptor-couples such as antigen-antibody [107] or streptavidin-biotin [81] could be interpreted by assuming that the reaction path involved several sequential barriers.

However, the conclusion that bond rupture could be adequately described with a two-parameter equation was disproved by the experimental finding that the force dependence of the off-rate was not always monotonous. Instead, the off-rate of so-called **catch-bonds** is lowest (and the bond lifetime is accordingly highest) for a value of the pulling force that is often of order of a few tens of piconewtons [72,108]. While much effort was made to elucidate the mechanisms of the so-called catch-bond phenomenon with molecular dynamics [109,110] and/or theoretical studies [106,111,112], no phenomenological equation gained a wide acceptance to replace Equation (4). Note also that some geometrical parameters, such as the force direction, are not accounted for by Bell’s equation [98,113].

Another problem was raised by quantitative studies made on biomolecular bonds: an implicit assumption underlying the aforementioned descriptions was that all studied bonds had reached an equilibrium state. However, this is not always warranted by experiments. Experimental studies performed with flow chambers rapidly evidenced the formation of transient attachments by molecules that were considered as strong binders, such as antibodies [107], integrins [114], or even streptavidin [115]. Indeed, this finding is fully consistent with the existence of multiple barriers on reaction paths [81]. Thus, it is not surprising that the stability of a molecular bond might be dependent on its history. As a minimal model, it was shown that the dependence of the off-rate on the bond age followed the following simple phenomenological law [116,117]:

k_off_(t) = k_off_(0)/(1 + α t) (5)

The dependence of the so-called strengthening parameter α on forces was approximated with an affine law for an antibody, and it was found independent of force for another antibody [117]. 

Thus, a complete description of the effect of forces on bonds would require to choose and measure more than one parameter. It is important to ask whether such a task would be warranted. It is, therefore, useful to discuss available information about the physiological relevance of the force dependence of ligand receptor bonds. In addition to a recent review [61], we shall briefly mention some important examples:

1. *Integrin mediated cell adhesion*. Integrins are important mediators of cell adhesion. A well-known peptide ligand of integrins (the RGD tripeptide) was bound to surfaces with DNA constructs of varying strengths. Cell adhesion required that the linkers be able to resist a force of at least 40 pN for 2 s [118].
2. *Signaling*. Similarly, the use of calibrated DNA tethers showed that notch signaling required that the strength of the ligand attachment to a surface be higher than 12 pN [118]. When T lymphocytes were made to contact ligands deposited on a probe connected to a BFP allowing real-time determination of interaction force, the triggering of a calcium rise indicative of cell activation was correlated to the application of a force of about 10 pN on the TCR [119]. Additionally, a force of 10 pN applied on TCRs with an optical trap was reported to strongly increase signaling [119]. Furthermore, some reports supported the hypothesis that TCR signaling required that the TCR-ligand bond behave as a catch bond [119,120]. More work is, however, needed to assess the generality of this requirement [96]. More recently, it was suggested that talin, a cytoskeleton associated molecules that acted as a force sensor, could filter mechanical noise as a consequence of specific mechanical properties [102].
3. *Ligand discrimination*. The capacity of membrane receptors to discriminate between a high number of potential ligands is an essential requirement for cell function. It is, therefore, important to emphasize that forces may play an important role in ligand discrimination. The capacity of B lymphocytes to select and extract antigens bound to surfaces was shown to rely on forces [121,122]. Forces were also reported to modulate the preference of αvβ3 integrin for fibronectin or vitronectin [123].

Thus, many important reports support the view that forces play an important role in cell function and warrant the search for a suitable set of parameters providing a sufficiently exhaustive and manageable account of the effect of forces on molecular interactions.

#### 3.3.4. Receptor Length and Conformational Dynamics

In addition to the thermodynamic, kinetic, and mechanical properties of intermolecular bonds that were thoroughly studied during decades, we shall now briefly mention some loosely defined properties of receptor molecules that may strongly influence cell function but are not usually directly included in the description of binding properties. At least three important processes are not fully accounted for by aforementioned properties of molecular interactions:

1. In addition to the molecular properties of binding sites, the **efficiency of the bond formation** between surface-attached molecules is strongly dependent on the **length and flexibility** of binding molecules so that binding sites might contact each other with a suitable orientation [91]. As a well-known example, the remarkable capacity of P-selectin molecules to tether rapidly flowing leukocytes in blood vessels is partly due to the unusually high length of the ligand-receptor couple (nearly 0.1 µm). Length and flexibility also play an important role in modulating multivalent attachments that are key to the **avidity** of interactions. Avidity might be loosely defined as some kind of “functional affinity” [116].
2. Receptor-mediated **signaling** is also highly dependent on the capacity of binding molecules to form multivalent attachments, since clustering of membrane molecules is often a key event of signal generation [61]. Indeed, an early step of the triggering of a signaling cascade is often the phosphorylation of a dedicated site on a molecule by a kinase brought close to this site. The **molecular reach** of molecules such as kinases or phosphatases is obviously dependent on the size and conformational flexibility of involved molecules [124]. Another important role of receptor length in the signaling process is the reorganization of intercellular contact zones as a consequence of the exclusion of bulky molecules from tight contact zones generated by short ligand-receptor couples. The importance of this mechanism was well demonstrated since the exclusion of the bulky CD45 phosphatase was found sufficient to trigger a signaling cascade in T lymphocytes [125,126,127].
3. The formation of **multimolecular assemblies** plays an important role in signal generation (as related to signalosome formation) as well as cell structural organization. **Conformation flexibility** may play a key role in this process [128] if bond formation results in the transient appearance of docking sites that may recruit additional molecules. Two points may be mentioned in this respect. (i) For the sake of simplicity, model systems used to study molecular associations most often rely on binary interactions with the underlying assumption that they are additive, which is not always warranted [129]. (ii) Recent progress in molecular dynamics may strongly improve our understanding of reaction paths and transient molecular states, which may increase our interest in ternary and multimolecular interactions.

## 4. Discussion and Conclusions: What Is the Take-Home Message?

The purpose of this review was to select a sufficient number of experimental and theoretical results to convince the reader that the parameters currently used to feed interactome databases might be insufficient to support optimal progress in the explanation and prediction of cell function. Indeed, while much effort was made to standardize the description of biomolecule interactions in order to make information optimally available [130,131] or facilitate retrieval by automated methods [132], there is no known procedure allowing to assess the utility of the more and more numerous pieces of information yielded by experimental studies and simulation of single bond formation and dissociation. 

While it was considered important to mention as noticeable examples some properties such as bond strengthening or the flexibility of binding molecules as potentially useful features to improve our understanding of cell function, if was obviously not feasible to present a clear-cut list of parameters that need to be added to interactome databases. However, the points detailed below might be worth to keep in mind.

A first question is to know whether there is an absolute need to feed interactome databases with quantitative parameters such as equilibrium or kinetic constants. Indeed, the seemingly obvious feeling that cell function is too complex to be liable to a description by qualitative information was formulated three decades ago in a *Nature* editorial [133]. Additionally, the complexity of molecular interactions might be considered as less amenable to qualitative descriptions than DNA sequence. However, as reviewed above, recent mathematical use of Boolean networks showed that important conclusions could be obtained by processing qualitative parameters, and it was emphasized that a strong limitation of quantitative treatments lies in the impossibility of knowing the precise state of a system [28]. Additionally, a time-series of qualitative set of data [65] might be sufficiently informative to unravel complex phenomena.

A second point is that different parameters may be needed to **predict** and to **understand** cell function. This is exemplified in a spectacular way by the progress of artificial intelligence, and this is remarkably illustrated by a recent breakthrough in the study of protein structure. While a quantitative account of intermolecular forces seems an obvious way of explaining protein structure, spectacular progress was obtained by using deep learning to take advantage of already known proteins to “guess” the structure of a new molecule [134]. 

Third, recent evolution of molecular dynamics may support the long-standing idea that a thorough description of cell behavior from basic molecular mechanisms should benefit from the study of intermediate scales (or so-called mesoscale phenomena) [93]. Indeed, the size limitation of systems that are liable to computer simulation for a reasonably long period of time was often successfully extended by taking use of coarse-grained descriptions that consisted of replacing individual atoms by weakly coupled subsystems [135] in addition to the development of smart sampling methods facilitating the study of rare events [84]. Clearly, different parameters may be required to account for different behavior scales.

## Figures and Tables

**Table 1:** Information given in representative public interactome database on P-selectin (CD62P) interaction with PSGL1 (P selectin glycoprotein 1)/CD162 ^1^.

Database	Website	CD162 Ligand	Affinity or Kinetic Constants	Catch Bond	Rolling Function	Nb of PUBLICATIONS Quoted
Biogrid	https://thebiogrid.org/ accessed on 7 December 2021	+	-	-	-	1
Dip	https://dip.doe-mbi.ucla.edu/dip/ accessed on 7 December 2021	+	-	-	-	0
HPRD	http://hprd.org/ accessed on 7 December 2021	+	-	-	-	3
IntAct	https://www.ebi.ac.uk/intact/ accessed on 7 December 2021	+	-	-	-	4
Mint	https://mint.bio.uniroma2.it/ accessed on 7 December 2021	-	-	-	-	-
String	https://string-db.org/ accessed on 7 December 2021	+	-	-	+	0
Uniprot	https://www.uniprot.org/uniprot accessed on 7 December 2021	+	+/-	-	+	11

^1^ Only immediately displayed information is shown. Bases are often interconnected and additional information can be obtained by following several sequential links.

## Data Availability

This review did not mention any unpublished information.

## Abbreviations

AFM: atomic force microscope, AP-MS: affinity purification—mass spectrometry, BFP: biomembrane force probe, LFC: laminar flow chamber, MD: molecular dynamics, MHC: major histocompatibility complex, pMHC: complex made by an oligopeptide p bound to an MHC-encoded molecule, PPI: protein–protein interaction, TCR: T cell receptor.
